# Assessment of hematological parameters in malarial suspected patients: Cross sectional study

**DOI:** 10.1016/j.parepi.2024.e00367

**Published:** 2024-07-15

**Authors:** Tanveer Khan, Abuzar Khan, Anis Khan, Farhad Badshah, Eliana Ibáñez-Arancibia, Patricio R. De los Ríos-Escalante, Bibi Maryam, Nimra Noor, Maria, Mostafa A. Abdel-Maksoud, Mohamed A. El-Tayeb, Arab Hussain

**Affiliations:** a Department of Zoology, Bacha Khan University Charsadda, Pakistan; b State Key Laboratory of Animal Biotech Breeding, Institute of Animal Science, Chinese Academy of Agricultural Sciences, Beijing 100193, China; c Department of Zoology, Abdul wali Khan University Mardan, Pakistan; d PhD Program in Sciences mentioning Applied Molecular and Cell Biology, La Frontera University, Temuco, Chile; e Laboratory of Engineering, Biotechnology and Applied Biochemistry – LIBBA, Department of Chemical Engineering, Faculty of Engineering and Science, La Frontera University, Temuco, Chile; f Department of Biological and Chemical Sciences, Faculty of Natural Resources, Catholic University of Temuco, Temuco, Chile; g Nucleus of Environmental Sciences, Faculty of Natural Resources, Catholic University of Temuco, Temuco, Chile; h Botany and Microbiology department, College of Science, King Saud University, Saudi Arabia

**Keywords:** Hematology, Plasmodium, Parameters, Malaria

## Abstract

**Background:**

Malaria is a Zoonotic disease, worldwide in distribution and caused by different species of plasmodium. It is a major cause of sickness and mortality in developing countries including Pakistan. This study was carried with the aim to find out the prevalence of malaria and to aware the people about this disease.

**Methods:**

The study was carried out in district charsadda. 120 blood samples were collected from suspects both male and female, during the period of March 2022 to September 2022 and were analyzed for CBC and for Microscopic examination.

**Results:**

Out of these 120 samples 12(10%) were found positive and 108(90%) were negative. The prevalence of malaria was more in the month of June and July. The infection was high in male (13.3%) as compared to female (6.6%). The prevalence was more in rural areas 8(13.3%) than in urban areas 4(6.6%).

**Conclusion:**

The Hemoglobin, Hematocrit, Platelets and Red Blood Cells were found more affected in positive samples as compared to other parameters. The present study will help the malarial control programs to focus on rural areas. The *Plasmodium vivax* is more common in the study area.

## 1 Introduction

Malaria, a name coined by Italians in 1753, comes from the Italian words ‘mala’ (bad) \and ‘aria’ (air), reflecting the belief that the disease was caused by noxious fumes from marshy terrain (Boucher et al., 2018). The roots of malaria can be traced back to the earliest civilizations, as evidenced by descriptions of the disease in ancient Chinese medical texts dating to 2700 BCE, as well as the Ebers Papyrus from roughly 1500 BCE (Cox, 2010). As the foremost parasitic infection on the planet, malaria poses a major challenge to global health, with developing countries like Pakistan bearing the brunt of its impact in terms of illness and death (Khan et al., 2018). Malaria is a serious infectious disease that easily spreads through populations, with cerebral malaria, caused primarily by *P. falciparum*, representing the most perilous form of the disease that can lead to a life-threatening complications (khan et al., 2013). Traditionally, four Plasmodium species *P. falciparum, P. vivax, P. ovale, and P. malariae* have been recognized as the culprits behind human malaria infections, but there is now evidence that *P. knowlesi*, which normally affects macaque monkeys, can also infect humans (Collins, 2012). (See Table 1, Table 2, Table 3, Table 4.)

**Table 1:** Age wise prevalence of Malaria.

Age Group	Total Sample	Positive	Negative
1-----20	23	01(4.3%)	22(95.6%)
21-----40	46	07(15.2%)	41(89.1%)
41-----60	37	03(8.1%)	34(91.8%)
61-----80	14	01(7.1%)	13(92.8%)
**Total**	**120**	**12**	**108**


Age wise prevalence was also detected in the people of different age groups in district Charsadda. Total 23 samples were collected from age group 1–20 years out of 23 samples 1 (4.3%) were positive and in age group 21–40 years total 46 samples were collected in which 7(15.2%) were found positive while in age group 41–60 years out of 37 samples 3(8.1%) were positive. The samples collected from age group 61–80 years were 14 in which 1(7.1%) were found positive.

**Table 2:** Hematological parameter and WHO value.

Variables	WHO cut-off level	Male	Female
Hemoglobin(Hb)	<13 g/dL& < 12 g/dL	Positive = **8**	Positive = **4**
Hematocrit(Hct)	<41%& < 36%		
Red Blood Cell(RBC)	<4.2 × 10^6^/cmm & < 3.6 × 10^6^/cmm		
Mean corpuscular volume(MCV)	<80 fL		
Mean Corpuscular Hemoglobin(MCH)	<27 pg		
Mean Corpuscular Hemoglobin Concentration(MCHC)	<32 g/dL		
Platelet count	<4.0 × 10^3^/cmm

**Table 3:** Male Hematological Changes.

Variables	WHO Value	WHO cut-off value	Detected Males	Total
Hemoglobin(Hb)	≥13 g/dl	<13 g/dL	6	8
Hematocrit(Hct)	≥41%	<41%	5	8
Red Blood Cells(RBCs)	≥4.2 × 10^6^/cmm	<4.2 × 10^6^/cmm	4	8
Mean Corpuscular volume(MCV)	≥80 fL	<80 fL	3	8
Mean Corpuscular Hemoglobin (MCH)	≥27 pg	<27 pg	2	8
Mean Corpuscular Hemoglobin Concentration(MCHC)	≥32 g/dL	<32 g/dL	0	8
Platelet count	≥4.0 × 10^3^/cmm	<4.0 × 10^3^/cmm	1	8

**Table 4:** Female Hematological Changes.

Variables	WHO Value	WHO cut-off value	Detected Females	Total
Hemoglobin(Hb)	≥12 g/dL	<12 g/dL	3	4
Hematocrit(Hct)	≥36%	<36%	1	4
Red Blood Cell(RBC)	≥3.6 × 10^6^/cmm	<3.6 × 10^6^/cmm	2	4
Mean corpuscular volume(MCV)	≥80 fL	<80 fL	0	4
Mean Corpuscular Hemoglobin(MCH)	≥27 pg	<27 pg	1	4
Mean Corpuscular Hemoglobin Concentration(MCHC)	≥32 g/dl	<32 g/Dl	0	4
Platelet count	≥4.0 × 10^3^/cmm	<4.0 × 10^3^/cmm	1	4

Among the six Eastern Mediterranean nations with high malaria transmission rates, Pakistan stands out as a country where virtually the entire population is at risk of contracting the disease. The prevalence of malaria, however, varies across different provinces and cities due to the diversity of climates (WHO, 2019). In 2017, the cumulative Annual Parasite Index (API) for Pakistan was 1.88, with the highest incidence of malaria reported in Khyber Pakhtunkhwa (30%), followed by Sindh (26.5%), Federally Administered Tribal Areas (FATA) (21.9%), Baluchistan (20.5%) and Punjab reported the lowest number of cases i.e., 1.1% (Qureshi et al., 2019).

## 2 Materials and methods

The present study was completed in a period of 7 months (March 2022 to September 2022). A total of 120 blood samples were collected from just symptomatic individuals and a proper questionnaire filled from each individual. The samples were collected at DHQ hospital Charsadda and DHQ Hospital Tangi Charsadda. Three ml of blood was drawn from each suspected person by using a sterilized syringe and transform into EDTA tube. The blood was then analyzed for CBC and Microscopic examination.

## 3 Results

A total of 120 blood samples were collected from only symptomatic patients at district Charsadda and this blood is tested through microscopy in laboratory. The data is also collected through proper questionnaire. Out of these 120 blood samples 12 samples were positive and 108 samples were negative. In this study hemoglobin, hematocrit, mean corpuscular volume, mean corpuscular hemoglobin, mean corpuscular hemoglobin concentration, platelets count, red blood cells count were determined by using hematology analyzer.

Out of 120 blood samples, 60 samples were taken from male and 60 from female. Out 60 males 8(13.3%) samples were found positive and 52(86.7%) were negative and out of 60 females 4(6.6%) samples were found positive and 56(93.4%) were negative.

### 3.1 Month wise prevalence of malaria in district Charsadda

Month wise data is collected from march2022 to September 2022. A highest number of malaria cases were found in June and July while the lowest number found in March.

**Table:** 

Months	Samples	Positive	Negative
**March**	10	0	10
**April**	10	0	10
**May**	20	2	18
**June**	30	3	27
**July**	30	4	26
**August**	10	2	8
**September**	10	1	9
**Total**	120	12	108

### 3.2 Hematological parameter and WHO value

The criteria for diagnosing anemia based on hemoglobin levels were 12 g/dl for females and 13 g/dl for males. Abnormal hematocrit values is below from 36% for females and below 41% for males. Normal RBC counts for males ranged from 4.2 to 5.8 × 10^6^/cmm, while for females they ranged from 3.6 to 5.6 × 10^6^/cmm. Anemia was indicated by RBC indices below MCV < 80 fl and MCH < 32 g/dl. Similarly, platelet counts below 150 × 10^3^/cmm were considered abnormal. After comparing the hematological profile of MP-positive patients with WHO values, the results were obtained.

### 3.3 Gender wise hematological changes

A total of 120 samples were collected from district Charsadda out of which 12 samples were found positive while 108 samples were negative. The positive samples were analyzed for hematological changes. MP-Positive male and female patient were have different hematological profile.

### 3.4 Male hematological changes

In male MP patients the hematology is effected like hemoglobin, hematocrit, red blood cells and platelets is below from the WHO value while the mean corpuscular hemoglobin concentration is not effected.

### 3.5 Female hematological changes

In female malarial patients the hemoglobin, red blood cells, hematocrit is affected while mean corpuscular volume (MCV) and mean corpuscular hemoglobin concentration is normal.

## 4 Discussions

Malaria is one of the major health problems and predominant of all parasitic infections in the world and is a major cause of sickness and mortality in developing countries with inclusion of Pakistan (Shah et al., 2016). According to the WHO 97% (approximately 150 million) of the Pakistani population is at risk of malaria, the transmission season is from September to November but high risk of malaria in KP is in the month of July and August (Khatoon et al., 2010). Malaria is usually associated with various degrees of reduced blood counts, and mild to moderate thrombocytopenia is a common association of malaria but it is rarely associated with hemorrhagic manifestations or a component of disseminated intravascular coagulation (Khattak et al., 2013). This survey was conducted from March 2022 to September 2022 to provide information on malaria prevalence in district Charsadda to find out the distribution of malaria in the people of district Charsadda. For this purpose a total of 120 blood samples were collected from district charsadda and this blood is tested through microscopy in laboratory. The data is also collected through proper questionnaire. Out of these 120 blood samples only 12(10%) samples were positive and 108(90%) samples are negative. These results is contrast with other provinces of Pakistan like Punjab province is very low; the prevalence ranged from 1.7% in Lahore to 5.5% in Bhakkar district. This stark divergence in prevalence rates can be attributed to the exceptional level of vigilance and care demonstrated by the residents of these cities, which serves to significantly mitigate the positivity rates. Sindh province reported increases in malaria prevalence in all areas, while Baluchistan province also reported an increase in the prevalence, The surge in malaria prevalence in Sindh and Baluchistan can be attributed to a lack of public awareness and adherence to preventive measures, as many people in the region are not taking adequate precautions and remain unaware of the disease's potential transmission 1990s (Rowland et al., 2002; Ladhani et al., 2002).

According to present study, 75% of cases have low Hb level. These result are in good agreement with WHO criteria. According to WHO 77% of our patients were anemic with regard to Hb values. Similarly, a study from Uganda had shown 76% prevalence of malaria parasitemia among severely anemic hospitalized patients in malaria endemic region. According to (Srichaikul, 1999), Anemia is one of the most common complications in malaria. The incidence of anemia in malaria was reported to be as high as 80%. These studies are in concordance with this study. The Present study show that 12–25% of cases are associated with thrombocytopenia as compared to much higher frequencies like 34.4% and 34% (Tan et al., 2008).

The Present study shows 50% cases with low RBC count in both male and female which is in accordance to 50.8% in *P. vivax* (Agravat and Dhruva, 2010). Anemia can be attributed to the destruction of red blood cell, splenic removal of parasitized and non-parasitized RBCs and dyserthropoiesis and engulfment in reticulo-endothelial system (Makani et al., 2011).

The effect of malaria on MCV, MCH, MCHC was no or little which is related with study performed by (Sirak et al., 2016). According to (Kotepui et al., 2014) HCT, MCV and MCHC were found significantly increased in malaria infected patients than that of controls in a study conducted in Thailand Myanmar border. Hematological parameters in this study were used to determine anemia induced by malarial parasitic infestation.

## 5 Conclusion

The Present study shows that *P. vivax* is more common in the study area as compared to other species. In present study the malaria infection is more in male as compared to female because male mostly have more outdoor activities as compare to female and also they do works in marshy places. The prevalence of malaria is more in rural area as compare to urban area because in rural areas most people don't have such money to afford expensive mosquito sprays or repellents. The hematological parameters is more effected in female as compared to male. The prevalence of malaria in the month of June and July is more as compared to other months because the summer season begins so most people start their outdoor activities and its suitable time for mosquito to bite person easily as compare to winter season.

**Recommendations:**

• Houses are sprayed before the season of greatest infection. This prevents malaria from being spread.
• Draining areas that could be used the breeding grounds.
• To educate people about Prevention, Risk factors through seminar, pamphlet, radio, media, newspaper etc.

## Funding

The present study was financed by project MECESUP UCT 0804, May 2023 and Project RSPD2024R678, 10.13039/501100002383King Saud University, Riyadh, Saud Arabia.

## Disclosure statement

In the course of preparing this work, the authors employed ChatGPT (Free Research Review, OpenAI) to rephrase or translate brief sections or sentences. Subsequently, following the utilization of this tool/service, the authors carefully examined and revised the content as necessary, assuming complete responsibility for the publication's substance.

## CRediT authorship contribution statement

**Tanveer Khan:** Conceptualization, Data curation, Formal analysis, Funding acquisition, Investigation, Methodology, Project administration, Resources, Software, Supervision, Validation, Visualization, Writing – original draft, Writing – review & editing. **Abuzar Khan:** Conceptualization, Formal analysis, Methodology, Writing – original draft. **Anis Khan:** Conceptualization. **Farhad Badshah:** Conceptualization, Funding acquisition, Investigation, Project administration, Resources, Supervision. **Eliana Ibáñez-Arancibia:** Conceptualization, Funding acquisition, Project administration. **Patricio R. De los Ríos-Escalante:** Conceptualization, Funding acquisition, Investigation, Project administration. **Bibi Maryam:** Conceptualization, Formal analysis, Writing – original draft. **Nimra Noor:** Conceptualization, Investigation, Resources, Writing – original draft. **Maria:** Conceptualization, Formal analysis, Writing – original draft. **Mostafa A. Abdel-Maksoud:** Funding acquisition, Project administration. **Mohamed A. El-Tayeb:** Funding acquisition, Project administration. **Arab Hussain:** Conceptualization, Data curation, Investigation, Software, Supervision, Validation, Writing – original draft.

## Declaration of competing interest

There is no competing interests among any of the authors in the study.
